# Transformation method of Φ-OTDR optical fiber strain and tunnel liner strain and its application in tunnel safety monitoring

**DOI:** 10.1038/s41598-026-43749-5

**Published:** 2026-03-17

**Authors:** Kai Cao, Ziliang Xie, Feicong Zhou, Xibao Wang, Jiabin Tang, Zhijie Wang

**Affiliations:** 1 State Key Laboratory of Disaster Reduction in Civil Engineering, Tongji University, Shanghai, 200092 China; 2 Department of Geotechnical Engineering, Tongji University, 1239 Siping Road, Shanghai, 200092 China; 3 Zhejiang Development & Planning Institute, Hangzhou, 310030 China; 4 College of Civil Engineering, Fuzhou University, Fuzhou, 350108 China; 5 Sichuan Communication Surveying & Design Institute Co., Ltd., Chengdu, 610017 Sichuan China; 6 Chengdu Land-Sub Technology Co., Ltd., Chengdu, 610051 Sichuan China; 7 School of Civil Engineering, Southwest Jiaotong University, Chengdu, 610031 Sichuan China

**Keywords:** Distributed optical fiber, Φ-OTDR sensing technology, Tunnel safety monitoring, Strain calibration scheme, Automatic monitoring, Engineering, Materials science

## Abstract

A distributed optical fiber stress and strain monitoring system (DSS) is developed based on Φ-OTDR sensing in this study. To apply the system to tunnel monitoring, dynamic and static tests of concrete beams are proposed to calibrate the relationship between Φ-OTDR strain and structural strain. The development trend of Φ-OTDR strain accumulation over time under the influence of the external environment is *ε*_*fiber, t*_=(0.023 ± 0.001)·*t*+(0.1425 ± 1.1795) through static tests. Combined with dynamic tests, the transformation relationship between Φ-OTDR strain and structural strain is *ε*_*struc*_ = 1.263·*ε*_*fiber*_*-*0.029·*t* + 0.708. The accuracy of the transformation method and the feasibility of Φ-OTDR fiber in structural strain monitoring are verified by tunnel field monitoring test. Finally, a tunnel safety warning platform is established based on Φ-OTDR technology. The calculation program and safety threshold library of monitoring indexes are implanted into the platform, making the system automatically judge and alarm the tunnel safety, thus ensuring the tunnel construction safety intelligently.

## Introduction

There are many uncertain hazards in tunnel construction because of the complex geology^1,2^. Precisely, tunnel monitoring can assess and predict the risk of tunnel construction, and then take disaster prevention and control measures in advance to avoid construction accidents^3–5^. Traditional equipment can meet the tunnel safety monitoring, but requires technical personnel to operate the equipment^6^. Many companies have developed automatic monitoring equipment and intelligent monitoring systems for tunnel construction with the advancement of technology^7–9^. Li et al.^10^ innovatively proposed a multi-layer deformation intelligent monitoring system for salt rock tunnel inverted arch by combining laser horizontal deformation monitor and optical fiber monitor. Scholars^11,12^ realized early crack detection and accurate crack width measurement based on fiber Bragg grating sensing technology. Li et al.^13^ used FBG strain sensor to monitor frost heaving strain of liner structure in extremely cold and high altitude area. Chai et al.^14^ used distributed optical fiber sensing technology to monitor the strain information of the structure, and found that the optical fiber strain characteristics can reflect the reinforcement effect of grouting on the tunnel. Li et al.^15^ developed a microseismic sensor based on optical fiber interference technology, which can monitor the whole process of tunnel surrounding rock from micro cracks to macro damage. In summary, the existing tunnel automation monitoring technology is diverse, but the complete set of technologies that are truly applied to tunnel construction monitoring and can realize safety warning are still few.

Notably, distributed optical fiber is widely used in tunnel automatic monitoring, and has many monitoring functions^16–18^. In addition, Monsberger and Lienhart^19^ found that optical fiber sensors are slightly superior to vibrational chord sensors in terms of measurement sensitivity, signal transmission distance, anti-electromagnetic interference ability, and dynamic response. However, it is necessary to wrap a special protective layer outside the optical core to prevent the optical fiber failure induced by construction when the newly developed distributed optical fiber is applied to tunnel monitoring. At this time, the deformation of the optical core lags behind the deformation of the protective layer, which leads to the inconsistency between the optical fiber strain and the tunnel structure strain^20–22^. Therefore, to make the new optical cable better applied to tunnel safety monitoring, the accurate transformation method between optical fiber strain and tunnel liner strain is critical.

To achieve high-precision measurement of multi-core fiber, the calibration process is an important step in the development of optical fiber sensors^23–25^. Hong et al.^26^ proposed an analytical model for analyzing the strain transfer mechanism between the FBG sensor and the measured geogrid, with a maximum relative error of 8.2%. Liu et al.^27^ established the correlation between packaging materials and optical fibers based on the strain transfer theory. More, the relationship between the central wavelength of the fiber and the deformation and temperature is evaluated by bending and temperature calibration tests. Tan et al.^28^ proposed a high-precision calibration method for fiber Bragg grating strain sensing based on an optical lever. Heilmeier et al.^29^ used neutron diffraction to calibrate fiber Bragg gratings for strain measurement inside cast aluminum. The existing optical fiber strain calibration methods are diverse, but the optical fiber strain calibration method for tunnel liner strain monitoring has not been studied.

The optical fiber has diverse monitoring functions, so it is of great significance to apply the newly developed optical cable to tunnel safety monitoring accurately. Notably, the calibration method for optical fiber strain and the safety early-warning system for lining monitoring are of critical importance. This study takes the developed Φ-OTDR distributed optical fiber stress and strain monitoring system (DSS) as an example. To apply the system to tunnel safety monitoring, the dynamic and static tests of concrete straight beams are proposed and used to calibrate the relationship between Φ-OTDR strain and structural strain. Notably, the accuracy of the strain transformation method and the feasibility of Φ-OTDR fiber in tunnel structural strain monitoring are verified by tunnel field monitoring test. Furthermore, an automatic monitoring and early warning platform for tunnel safety is established based on Φ-OTDR optical fiber technology. The calculation program and safety threshold library of the monitoring indexes are implanted into the platform, which makes the system automatically judge and alarm the tunnel safety, so as to intelligently guarantee the tunnel construction safety.

## Φ-OTDR distributed optical fiber monitoring principle

As a kind of optical fiber sensing technology, Φ-OTDR technology senses the intensity and phase changes of backward Rayleigh scattering light induced by external factors (vibration, temperature, strain, etc.) by detecting backward Rayleigh scattering light in optical fiber.

### Principle of Φ-OTDR sensing technology

Both the Φ-OTDR system and the traditional OTDR system inject a pulse light into the fiber under test to detect the backward Rayleigh scattering light signal in the time domain. Different from the OTDR system, the Φ-OTDR system uses a narrow linewidth, high frequency stability light source, which ensures that the light injected into the fiber is highly coherent, while ensuring the stability of the backward Rayleigh scattering curve.

**Fig. 1:**
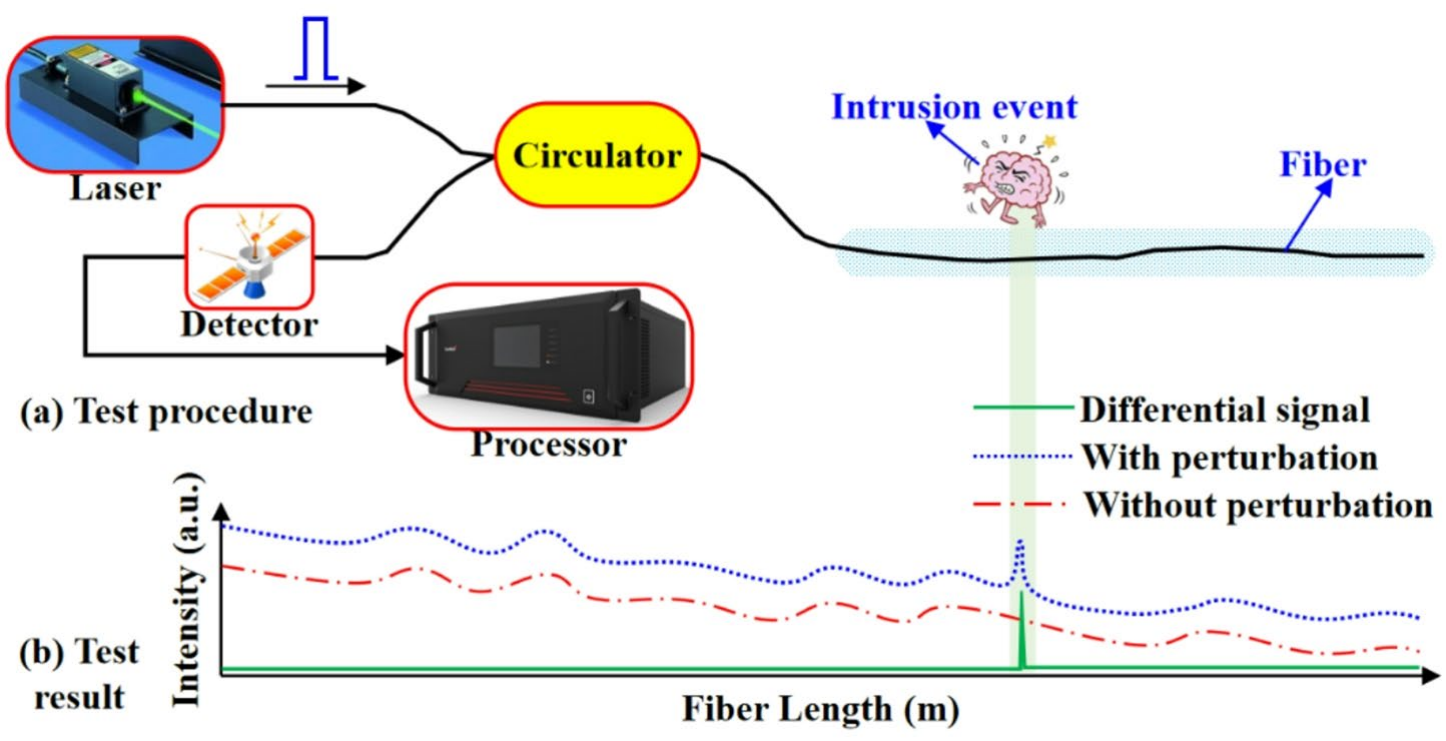
Principle of Φ-OTDR sensing technology.

Figure 1 is a schematic diagram of the Φ-OTDR sensing principle. The continuous laser output from the narrow linewidth laser is modulated by the modulator to a high extinction ratio optical pulse, and then enters the sensing fiber through the circulator. The backward Rayleigh scattering light at different positions of the fiber will interfere with each other within the pulse width, and the backward Rayleigh scattering light after interference enters the detector through the circulator for detection. The electrical signal output by the detector represents the intensity information of the backward Rayleigh scattering light along the length of the fiber^30^. Assuming that the laser frequency and phase output by the laser source are stable and that the fiber is not affected by disturbance, the intensity of the backward Rayleigh scattering light will remain stable, as shown in Fig. 1b. The refractive index of the fiber at the external intrusion position will change due to the elasto-optical effect, which will lead to the change of the phase of the backward Rayleigh scattering light. The interference intensity will change after the scattered light interferes with each other within the pulse width. And the intensity of Rayleigh scattering light remains unchanged in other locations without external intrusion.

### Φ-OTDR strain sensing mechanism

**Fig. 2:**
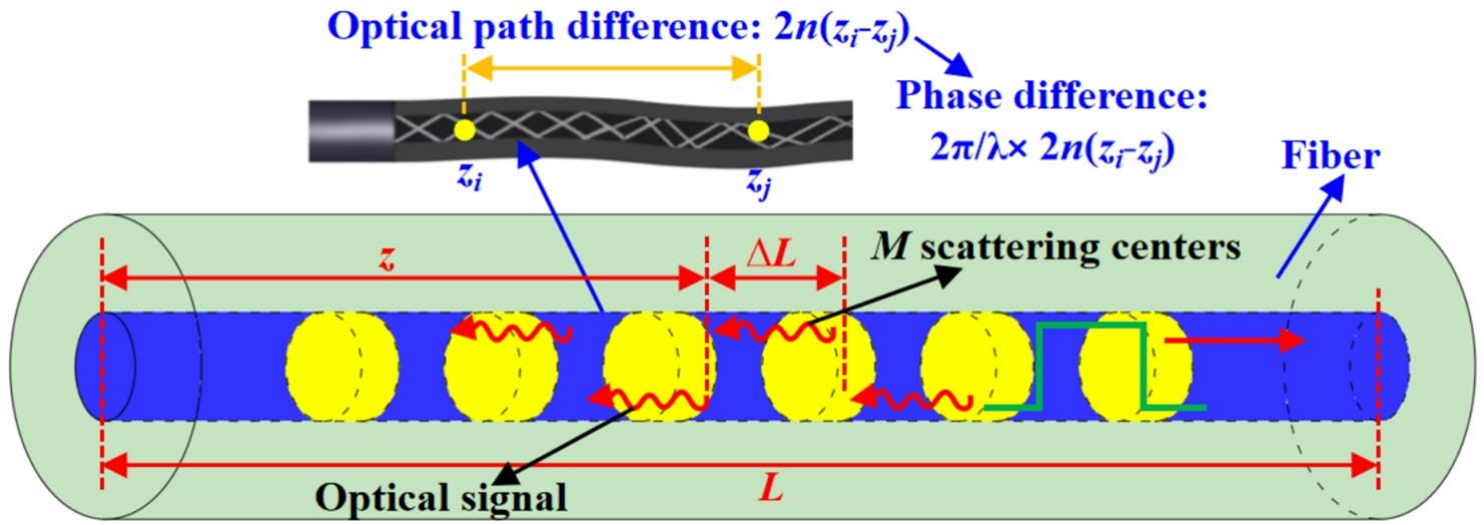
Φ-OTDR strain sensing mechanism.

The quantitative relationship between the phase (or intensity) of the backward Rayleigh scattering light and the strain can be obtained based on the 1-D Rayleigh scattering model^31^. As shown in Fig. 2, for two points with a fixed interval of $({z_i} - {z_j})$ on the fiber, the optical path difference is $2n({z_i} - {z_j})$ due to the round-trip process. According to the relationship between the phase difference and the optical path difference, the phase difference between two points is expressed as Eq. (1). Equation (2) can be obtained by differentiating both sides of Eq. (1). It can be seen that the phase variation is linear with the strain if the interval between two different positions is constant. Therefore, the strain can be determined by measuring the phase change of the backward Rayleigh scattering light between two points.

1 $${\varphi _{ij}}=\frac{{4\pi }}{\lambda }n({z_i} - {z_j})=\frac{{4\pi }}{\lambda }n{z_{ij}}$$

2 $$\Delta {\varphi _{ij}}=\frac{{4\pi }}{\lambda }(n+{C_\varepsilon }) \cdot {z_{ij}}\Delta \varepsilon$$

where ${z_i}$ and ${z_j}$ are the distances from two different positions on the fiber to the incident end of the laser. $\lambda$ is the laser center wavelength. *n* is the effective refractive index of fiber. $\Delta {z_{ij}}$ is the change of fiber length caused by strain. $\Delta \varepsilon$ is the amount of strain change. ${C_\varepsilon }$ is the refractive index strain coefficient.

Since the optical pulse has a certain width *W*, only the scattered light within *W*/2 width can have coherent superposition effect. The region where coherent superposition occurs is called the scattering region, and the length is set to $\Delta L$. Considering the scattering region of $z - z+\Delta L$, there are *M* scattering centers in the scattering region of $\Delta L$ (see Fig. 2). The amplitude of coherent superposition of backward Rayleigh scattering light generated in this region can be expressed by Eq. (3).

3 $$E(z)={E_0}\sum\limits_{{i=1}}^{M} {{a_i}{e^{j{\varphi _i}}}}$$

where ${E_0}$ is the initial amplitude of the incident light. ${a_i}$ is the scattering coefficient of Rayleigh scattering light at the *i*-th scattering center in the scattering region. ${\varphi _i}$ is the scattering phase of Rayleigh scattering light at the *i*-th scattering center in the scattering region.

It can be seen from the Eq. (3). that the interference results existing in each scattering region can be expressed by the sum of the scattered light wave functions in the region^30^. The Φ-OTDR signal intensity ($\Delta I$) can be expressed as Eq. (4).

4 $$\Delta I \approx 2E_{0}^{2}\sum\limits_{{i=1}}^{{M - 1}} {\sum\limits_{{j=i+1}}^{M} {{a_i}{a_j}\sin ({\varphi _{ij}})} } \propto 2E_{0}^{2}\sum\limits_{{i=1}}^{{M - 1}} {\sum\limits_{{j=i+1}}^{M} {{a_i}{a_j}\sin ({\varphi _{ij}})} } \Delta \varepsilon$$

### Optical fiber equipment based on Φ-OTDR technology

**Fig. 3:**
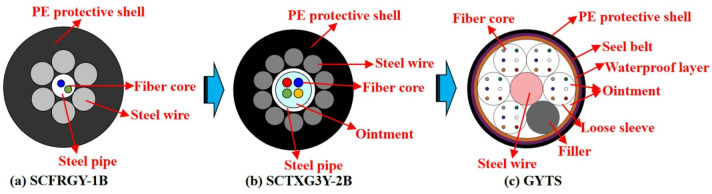
Development process of armored optical cable structure.

In this study, the GYTS distributed armored optical cable provided by Chengdu Land-Sub Technology Co., Ltd. is used. The spatial resolution, sampling interval, and dynamic range are 0.2 m, 5 GHz, and ± 3000 µε, respectively. The development process of the cable structure is shown in Fig. 3. The SCFRGY-1B cable will be broken due to excessive curvature or concrete grouting during installation, and the survival rate during construction is less than 20%. The steel pipe in the SCTXG3Y-2B optical cable is filled with ointment, so that the optical fiber can wriggle within a certain range when the optical fiber is stressed. However, the long-term allowable tensile force is only 200 N and the long-term survival rate is low. The loose sleeve in the GYTS cable has good toughness and high strength, and the center reinforced steel core helps the cable to be parallel and stretched, resulting in a cable survival rate of 92%.

**Fig. 4:**
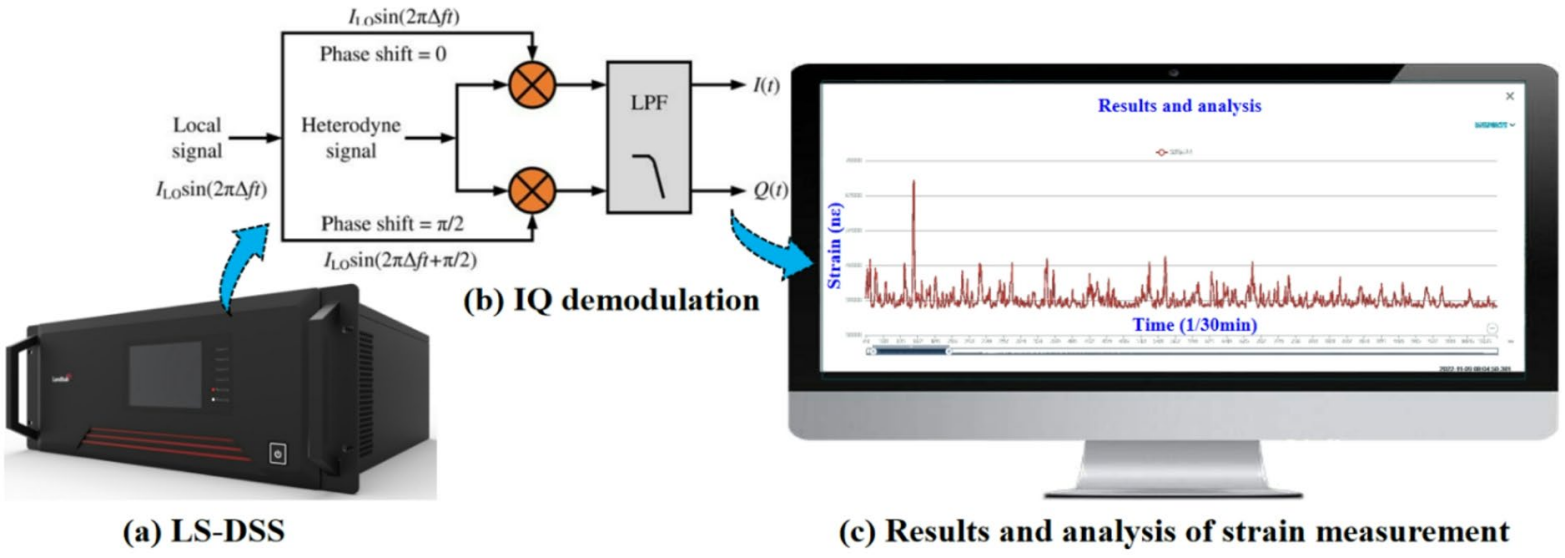
DSS strain data acquisition and analysis.

The Φ-OTDR dynamic nano-strain measurement device uses a distributed optical fiber stress and strain monitoring system (DSS). The device is a set of stress and strain monitoring system based on AIOT technology, which can realize distributed, long distance and precise positioning. DSS uses optical fiber sensing technology and intelligent signal processing algorithm to realize strain data acquisition and analysis (see Fig. 4). The algorithm mainly includes IQ demodulation algorithm^31^ and Φ-OTDR strain sensing mechanism related algorithm.

In this study, the center wavelength of the continuous light output by the narrow linewidth semiconductor laser is 1550.06 nm, the optical pulse width is 20 ns, the optical power is 20 mW, and the sampling rate is 5 GHz.

## Transformation scheme of Φ-OTDR strain and tunnel liner strain

Although the Φ-OTDR strain sensing mechanism is described in Sect. 2, it is found that the Φ-OTDR strain is inconsistent with the tunnel structural strain when it is applied to tunnel monitoring. The reasons for this are mainly divided into two categories. The main two reasons are as follows: firstly, the optical core has an outer protective layer, and the deformation of the optical core lags behind that of the PE protective layer. Secondly, the Φ-OTDR strain is affected by environmental factors, and strain accumulation also occurs in static state. To solve the difference of monitoring results between Φ-OTDR strain and tunnel structural strain, the test results of distributed armored optical cable and resistance strain gauge are compared, and the transformation relationship between them is discussed. Notably, different types of optical cables have different structures, resulting in different transformation relationship between Φ-OTDR strain and structural strain. The transformation scheme proposed in this study takes GYTS cable as an example.

### Transformation method of Φ-OTDR strain and structural strain

**Fig. 5:**
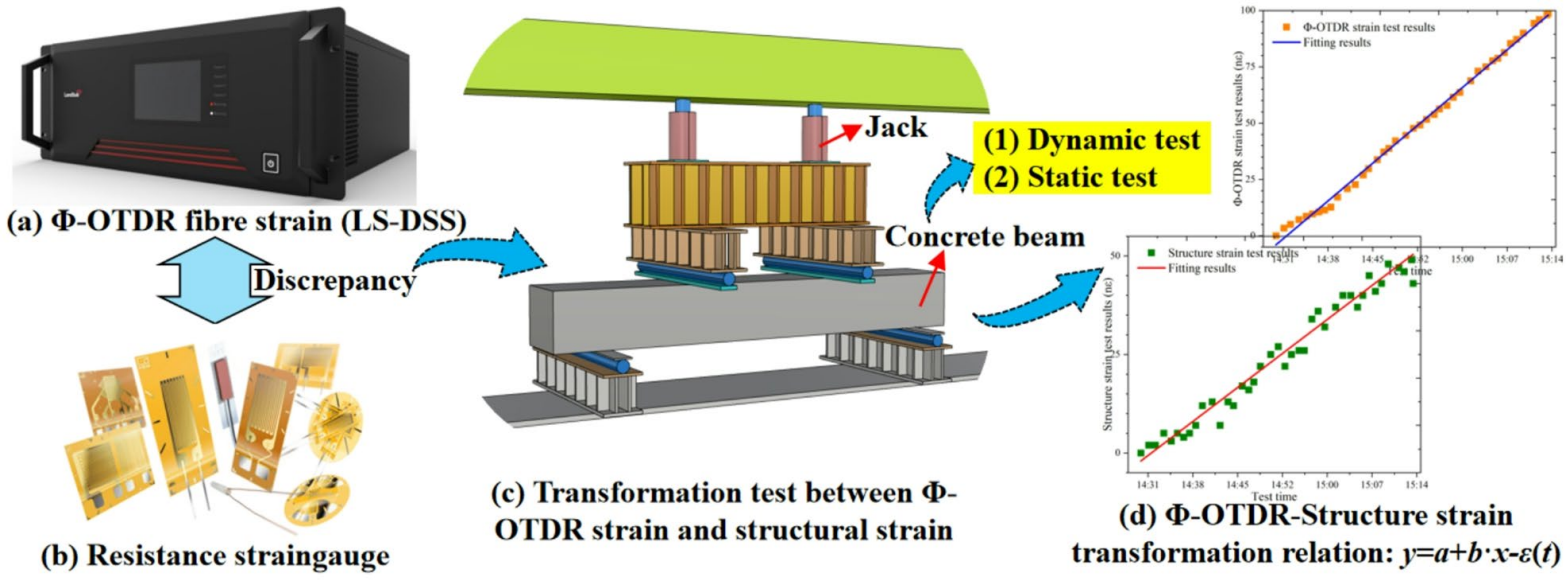
Transformation method of Φ-OTDR strain and structural strain.

In this study, the relationship between Φ-OTDR strain and structural strain is analyzed by bending test of concrete straight beam. The specific Φ-OTDR strain and structural strain transformation test includes the following steps (see Fig. 5).

Step 1, three concrete straight beams simulating tunnel structure need to be manufactured. The size and reinforcement ratio of concrete beams are the same as those of tunnels. Step 2, optical cable and resistance strain gauge are arranged on the lower surface of concrete beam. Then the concrete beam is placed on the test loading equipment (see Fig. 5c). Step 3, according to the straight beam bending test requirements in the specification^33^, the applied load is loaded according to 1 kN/min in the elastic range of the concrete beam, which is the dynamic test. Step 4, the test results of Φ-OTDR strain and structural strain are collected by DSS equipment and strain acquisition device without applying any load, which is the static test. Step 5, the transformation formula between Φ-OTDR strain and structural strain is derived, and then substituted into the dynamic and static test results to determine the correlation coefficient.

### Equipment for concrete straight beam bending test

**Fig. 6:**
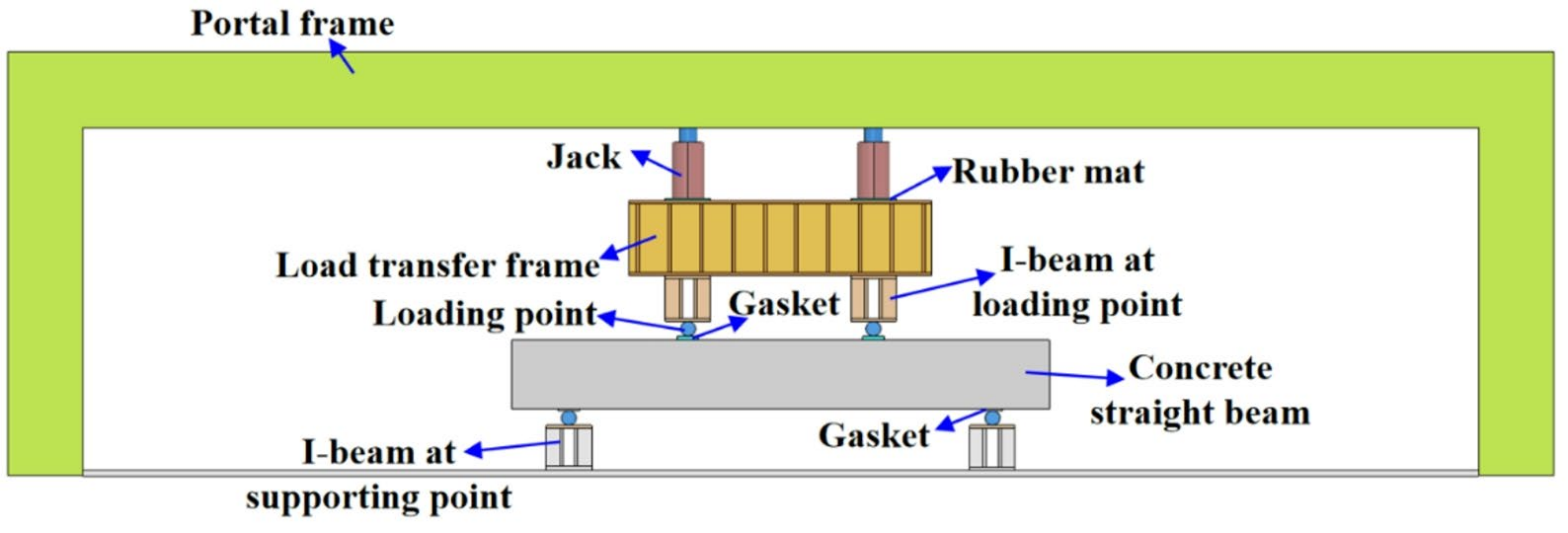
Equipment for concrete straight beam bending test.

The bending test equipment of concrete straight beam is mainly composed of portal frame, jack, load transfer frame, loading point and I-beam (see Fig. 6). The pressure generated by the jack makes the two loading points produce downward nodal force through the load transfer frame, which causes the bending deformation of the concrete straight beam.

### Manufacture of concrete straight beam

**Fig. 7:**
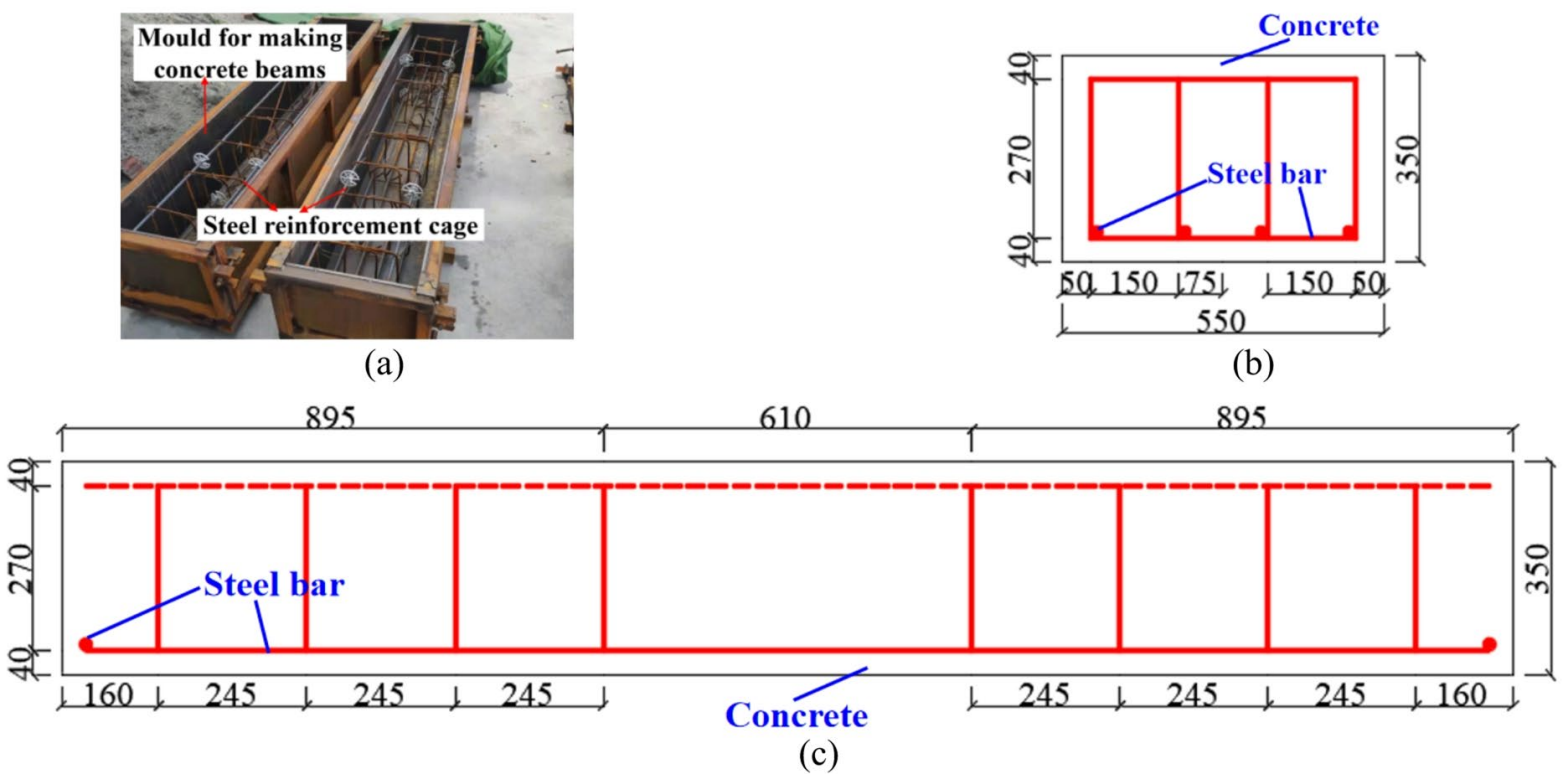
Size and reinforcement design of concrete straight beam specimens. (**a**) Straight beam mold, (**b**) Straight beam cross section, (**c**) Longitudinal section of straight beam.

The size of concrete straight beam specimen is determined to be 2400 mm × 550 mm × 350 mm considering the characteristics of tunnel liner and the requirements of specification^33^. Figure 7a shows the specimen mold and the Φ12@150 steel cage. The design of reinforcement distribution is shown in Figs. 7b,c. The concrete straight beam specimens are poured with C35 concrete. To reduce the uncertainty error of the test, three straight beam specimens are made.

### Layout scheme of the optical fiber and the strain gauge

**Fig. 8:**
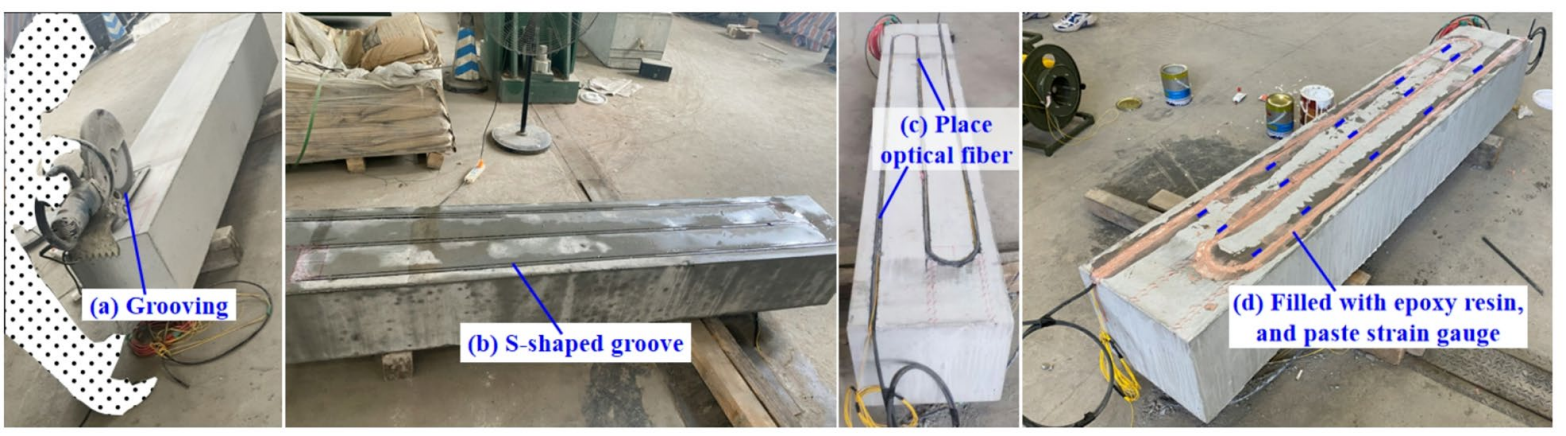
S-shaped groove cutting and fiber embedding method.

To coordinate the deformation of the optical fiber with the straight beam, the optical fiber is arranged on the surface of the specimen by slotting and embedding. Firstly, S-shaped grooves are cut on the lower surface of the straight beam, and the size of the grooves is 120% of the fiber diameter. Secondly, the optical fiber is installed in the S-shaped groove. Then, epoxy resin is used to fill the groove according to the experimental experience in reference^32^, so that the optical fiber and the straight beam are integrated (see Fig. 8).

**Fig. 9:**
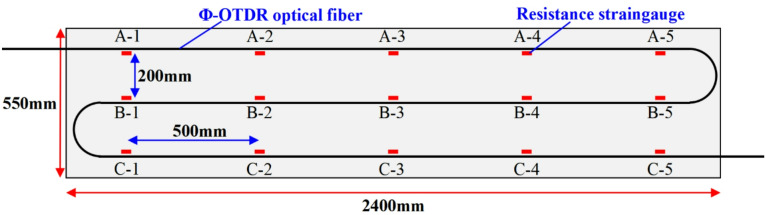
Layout scheme of the optical fiber and the strain gauge.

To obtain a large number of optical fiber and strain gauge monitoring comparison data, the distributed optical fiber measurement point spacing is reduced to 500 mm in the DSS setting. The strain gauge is arranged corresponding to the position of the optical fiber measuring point, and is also in an S shape. A total of 15 measuring points are arranged and numbered (see Fig. 9). The transverse spacing of the strain gauge is 200 mm and the longitudinal spacing is 500 mm.

### Loading scheme of concrete beam bending test

**Fig. 10:**
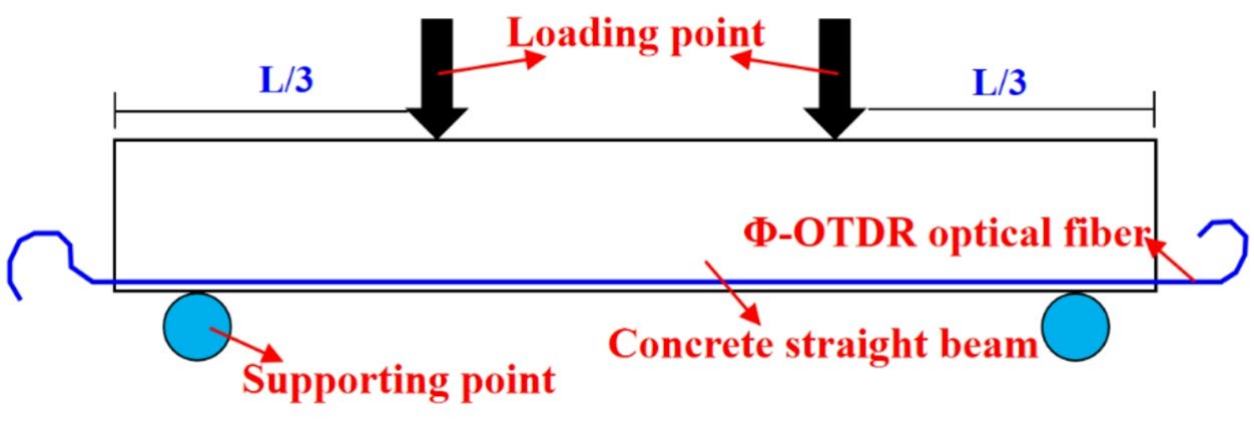
Trisection loading method.

To obtain a large amount of data, the dynamic test is to load the applied load according to 1 kN/min in the elastic range of the concrete beam. The test load is controlled within the limit state of normal use of concrete beams, which can realize the reuse of straight beams. According to the description of the straight beam bending test in the specification^33^, the dynamic test adopts the trisection loading method (see Fig. 10). The load of the concrete straight beam in the limit state is 85 kN by preloading. 60% (50kN) of the load is used as the upper limit of the dynamic test, and the load is loaded at 1 kN/min. The test data of Φ-OTDR strain and structural strain are collected by DSS equipment and strain acquisition device respectively.

**Fig. 11:**
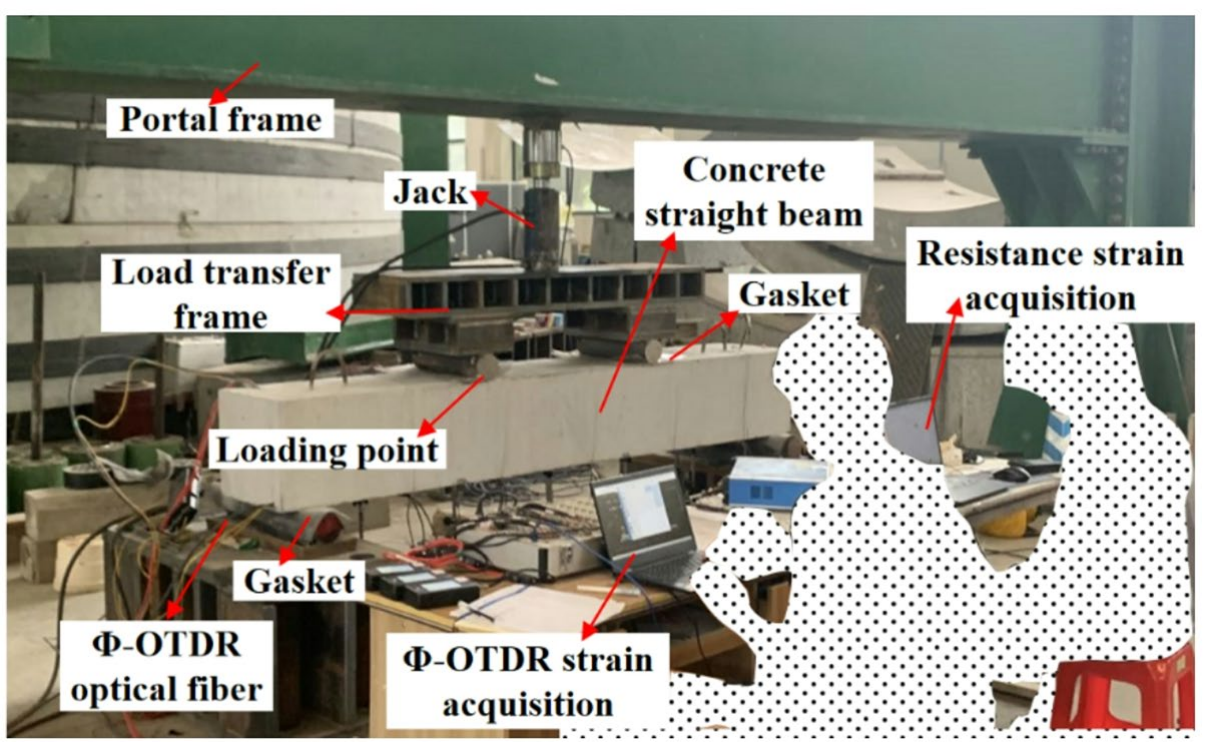
Concrete beam bending test scene.

The static test refers to the optical fiber strain and resistance strain test of the straight beam without any load for more than 30 min. The purpose is to increase the strain accumulation (∆*ε*(*t*)) caused by the fiber itself and the external environment on the transformation formula. The concrete beam bending test scene is shown in Fig. 11.

## Transformation test results of Φ-OTDR strain and structural strain

The data analysis process of strain transformation test is as follows: (1) The time history relationship of Φ-OTDR strain and resistance strain are summarized and fitted respectively. (2) Summarize and fit the time history relationship of Φ-OTDR strain in static test. (3) The transformation formula between Φ-OTDR dynamic strain and structural strain is derived, and then substituted into the dynamic and static test results to determine the correlation coefficient.

### Analysis of dynamic and static strain test results

**Fig. 12:**
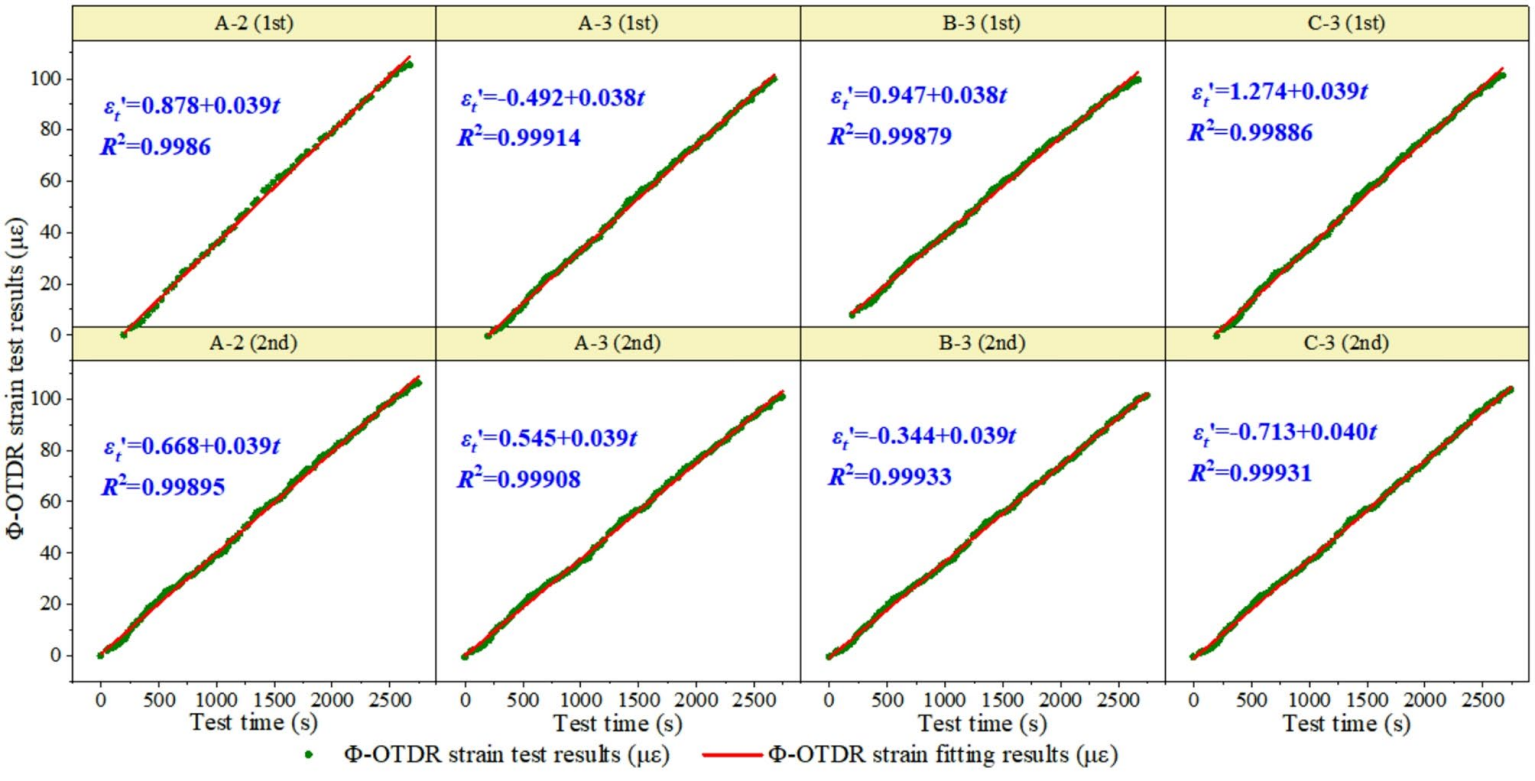
The time-history relationship of Φ-OTDR strain in dynamic test.

**Fig. 13:**
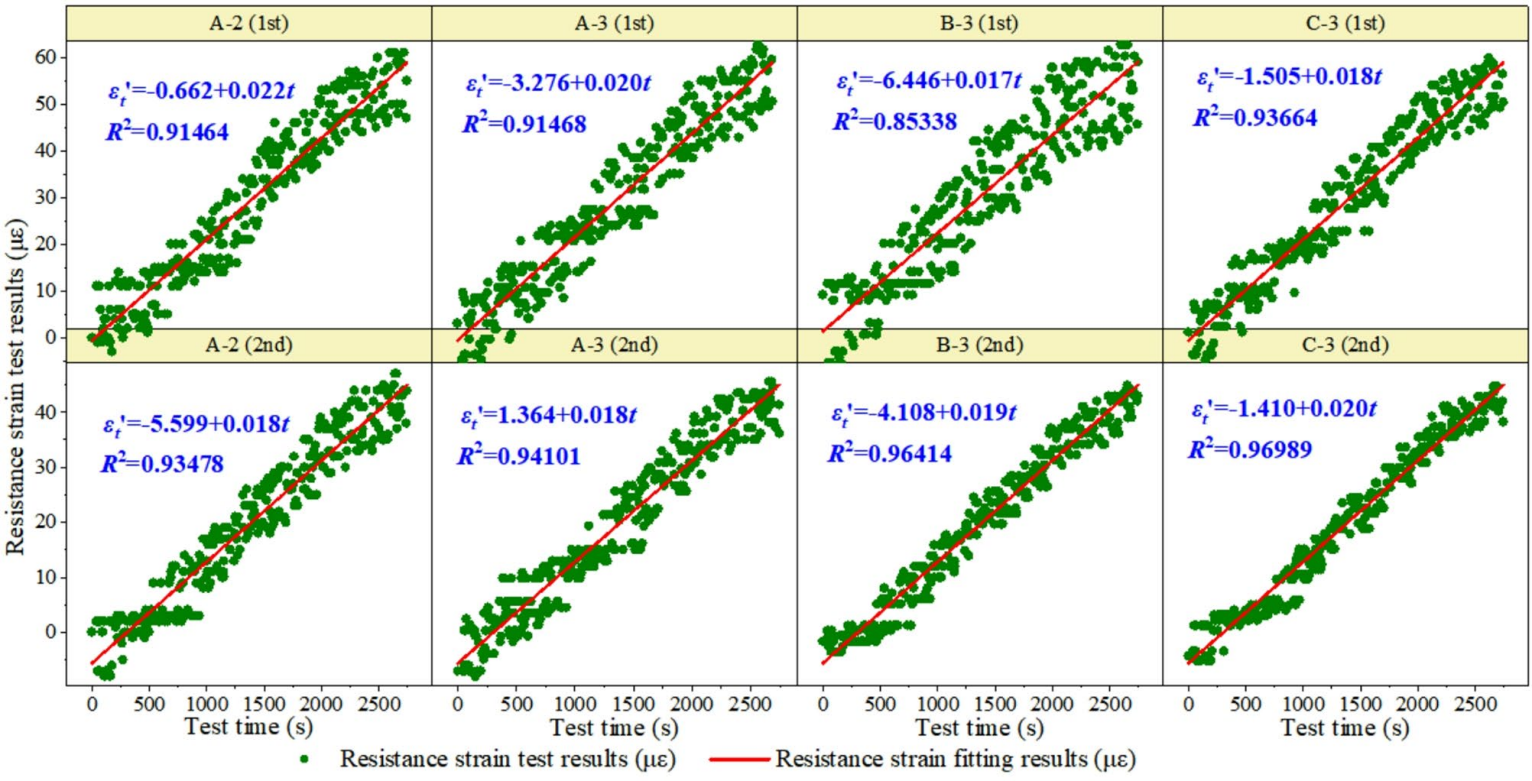
Time time-history relationship of resistance strain in dynamic test.

**Fig. 14:**
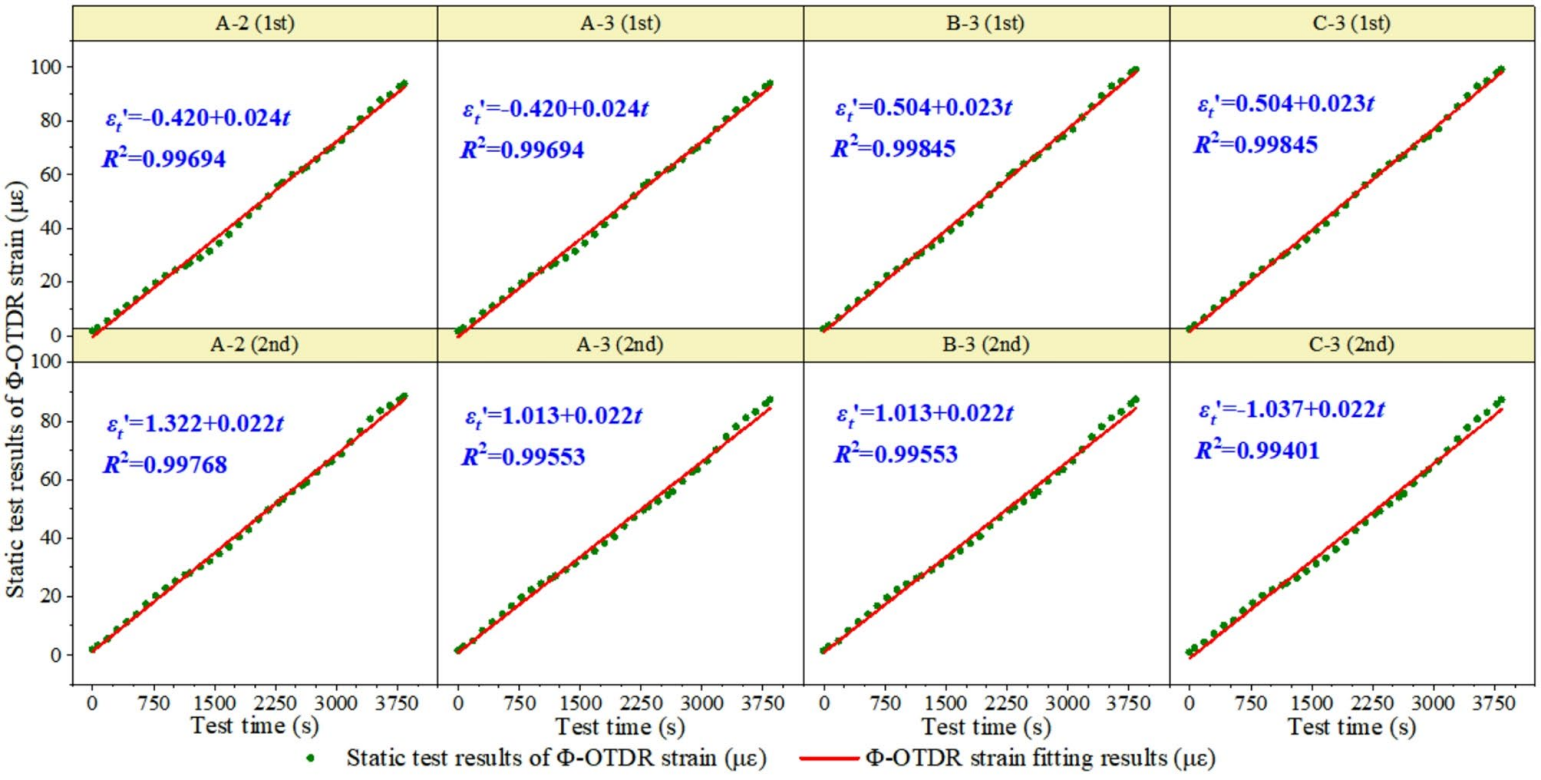
The time-history relationship of Φ-OTDR strain in static test.

The data statistics show that the test results of the measuring points at the middle position of the concrete beam are the best. Therefore, this study analyzes the two test results of the monitoring points A-2, A-3, B-3, and C-3 (see Figs. 12, 13 and 14). In the data processing process, one, two, three and other multiple polynomials, as well as power functions are used for fitting. It is found that the strain increases linearly with the increase of test load. The linear function is determined as the optimal fitting function of strain transformation relation according to the correlation coefficient R^2^. The strain-time relationship of Φ-OTDR obtained from the dynamic test is shown in Fig. 12. The slope range of Φ-OTDR strain-time history relationship is 0.038 to 0.040, and the intercept range is −0.713 to 1.274.

The resistance strain-time history relationship obtained from the dynamic test is shown in Fig. 13. The slope range of the resistance strain time-history relationship is 0.017 to 0.022, and the intercept range is −6.446 to 1.364.

The Φ-OTDR strain-time relationship in the static test is shown in Fig. 14. The slope range of Φ-OTDR strain-time history relationship in static test is 0.022 to 0.024, and the intercept range is −1.037 to 1.322. That is, the cumulative development trend of Φ-OTDR dynamic strain over time obtained from the static test is $\varepsilon _{{fiber}}^{t}=(0.023 \pm 0.001) \cdot t+(0.1425 \pm 1.1795)$.

### Transformation formula of Φ-OTDR strain and structural strain

The strain measured by GYTS fiber ($\varepsilon _{{fiber}}^{{}}$) is the sum of the strain caused by structural deformation ($\varepsilon _{{fiber}}^{ * }$) and external factors ($\varepsilon _{{fiber}}^{t}$) over time. The strain caused by structural deformation in the fiber ($\varepsilon _{{fiber}}^{ * }$) can be expressed by Eq. (5). The structural resistance strain time history relationship is expressed by Eq. (6).

5 $$\left\{ \begin{gathered} \varepsilon _{{fiber}}^{{}}=a \cdot t+b \hfill \\ \varepsilon _{{fiber}}^{t}=e \cdot t+g \hfill \\ \varepsilon _{{fiber}}^{*}={\varepsilon _{fiber}} - \varepsilon _{{fiber}}^{t}=a \cdot t+b - (e \cdot t+g) \hfill \\ \end{gathered} \right.$$

6 $${\varepsilon _{struc}}=c \cdot t+d$$

where ${\varepsilon _{fiber}}$ is the measured GYTS fiber strain (unit: µε). $\varepsilon _{{fiber}}^{t}$ is the cumulative strain caused by external factors over time. *t* is the accumulated time of optical fiber monitoring from the zeroing moment (unit: s). *a*, *b*, *c*, *d*, *e* and *g* are coefficients to be determined. These coefficients are determined by fitting the test results in Sect. 4.1.

The time *t* in Eq. (5) and Eq. (6) is offset to obtain the relationship between the structural resistance strain and the Φ-OTDR strain of the GYTS fiber, as shown in Eq. (7).

7 $$\left\{ \begin{gathered} {\varepsilon _{struc}}=k \cdot ({\varepsilon _{fiber}} - \varepsilon _{{fiber}}^{t})+Int=k \cdot ((a - e) \cdot t+b - g)+Int=c \cdot t+d \hfill \\ k=c/(a - e) \hfill \\ Int=c \cdot t+d - k \cdot ((a - e) \cdot t+b - g)=c \cdot (g - b)/(a - e)+d \hfill \\ \end{gathered} \right.$$

where ${\varepsilon _{struc}}$ is the structural strain (unit: µε). *k* is the slope. *Int* is the intercept.

It can be obtained that the slope range of the linear transformation formula of Φ-OTDR strain and structural strain is 1.059 to 1.467, and the intercept range is −6.948 to 1.860 by substituting the two test data into Eq. (7) (see Table 1). That is, the linear transformation relationship between Φ-OTDR strain and structural strain is Eq. (8).

8 $${\varepsilon _{struc}}=(1.263 \pm 0.204) \cdot ({\varepsilon _{fiber}} - \varepsilon _{{fiber}}^{t})+(0.9165 \pm 5.7645)$$

**Table 1:** Coefficients of the strain transformation formula derived from different measurement points.

	Measurement points	*k*	*Int*
1 st test	A-2	1.467	−2.566
	A-3	1.429	−3.173
	B-3	1.133	−6.948
	C-3	1.125	−2.371
2nd test	A-2	1.059	−4.907
	A-3	1.059	1.860
	B-3	1.118	−2.591
	C-3	1.111	−1.770

The static test result ($\varepsilon _{{fiber}}^{t}=(0.023 \pm 0.001) \cdot t+(0.1425 \pm 1.1795)$) is substituted into the Eq. (8) to obtain the modified transformation formula, as shown in Eq. (9).

9 $${\varepsilon _{struc}}=(1.263 \pm 0.204) \cdot {\varepsilon _{fiber}} - (0.029 \pm 0.006) \cdot t+(0.708 \pm 7.495)$$

The final transformation formula is ${\varepsilon _{struc}}=1.263 \cdot {\varepsilon _{fiber}} - 0.029 \cdot t+0.708$ after averaging the coefficients.

## Verification of Φ-OTDR strain monitoring results in tunnel

To further explore the application effect of Φ-OTDR distributed optical fiber in tunnel liner strain monitoring, Φ-OTDR optical fiber is applied to the safety monitoring of Ma’anshan tunnel (located in Sichuan, China). The optical fiber and vibrational chord strain gauges are arranged on the same tunnel section. Then the monitored Φ-OTDR strain is transformed by the transformation formula in this study to obtain the structural strain. Compared with the monitoring results of vibrational chord strain gauge, the similarity rate of the monitoring results of the two products is used to verify the accuracy of the transformation formula proposed in this study and the feasibility of the application of Φ-OTDR optical fiber in tunnel monitoring.

### Layout of optical cable and vibrational chord strain gauge

#### Layout of Φ-OTDR optical cable

**Fig. 15:**
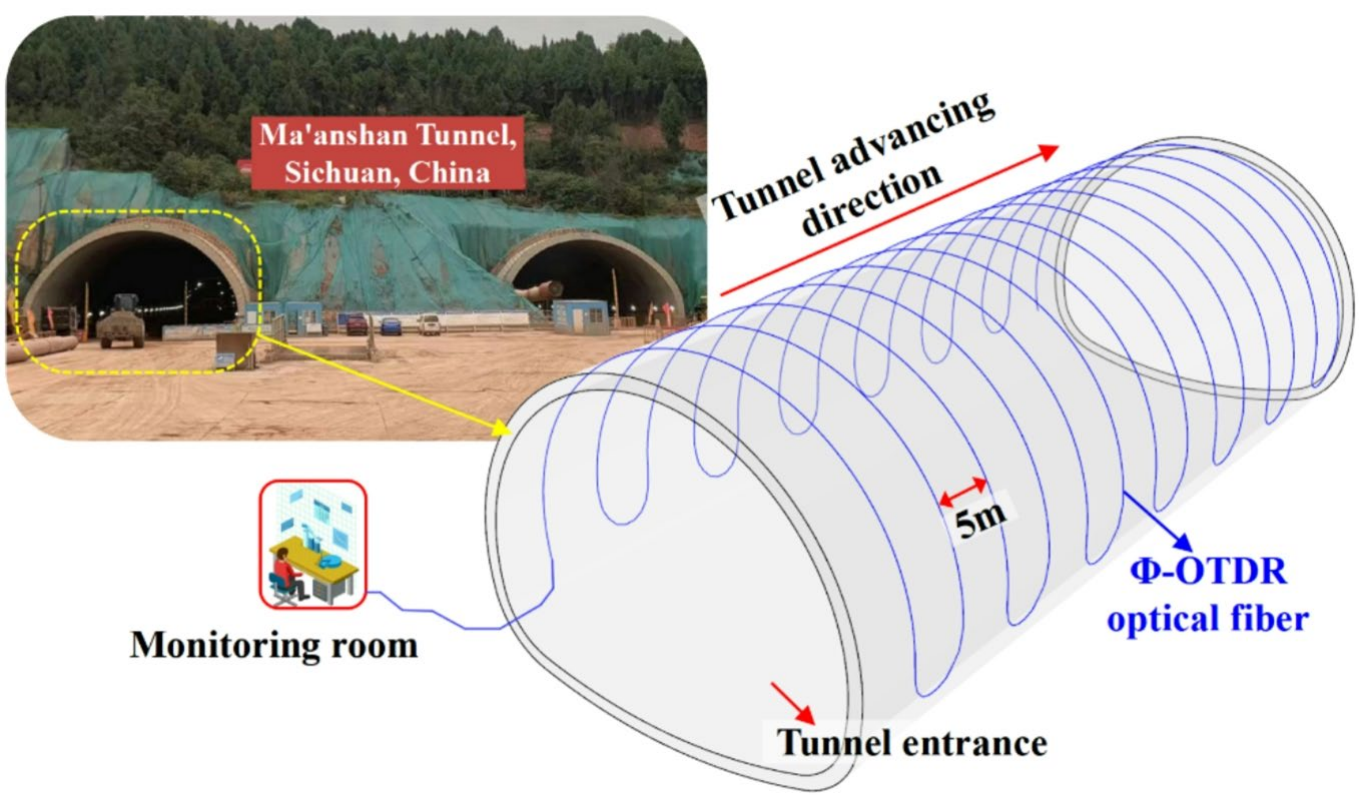
Optical cable laying scheme for liner strain monitoring of Ma’anshan tunnel.

Figure 15 shows the optical cable laying scheme for liner strain monitoring of Ma’anshan tunnel. The optical cable covers the tunnel vault, hance, arch springing and sidewall. The longitudinal spacing of the optical cable along the tunnel is 5 m. The optical cable is arranged in U shape, and the arc layout is adopted at the foot of the tunnel wall. A monitoring room is set up at the tunnel entrance for monitoring result analysis and early warning information feedback.

**Fig. 16:**
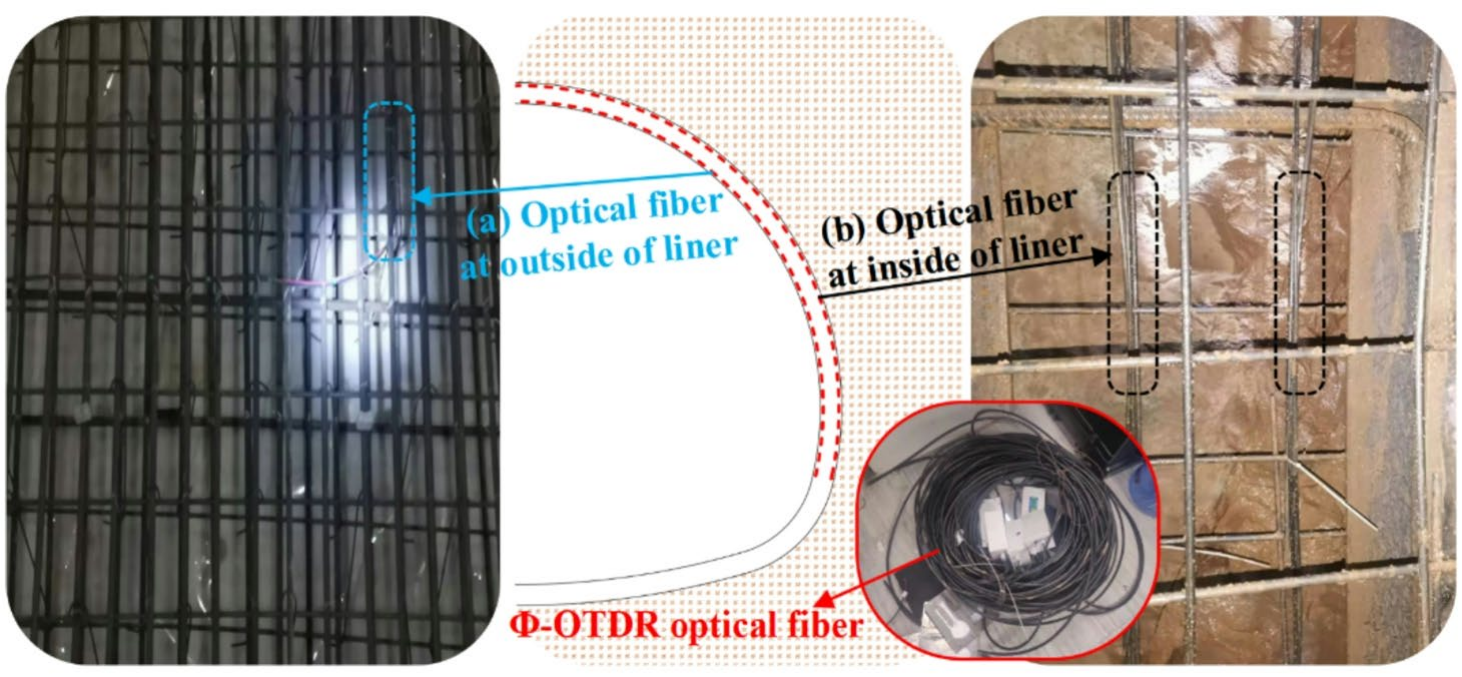
Layout of Φ-OTDR optical cable.

The optical cable used for verification is laid in the liner. The optical cable is fixed to the main reinforcement with laces after the welding of the secondary liner steel frame is completed. Notably, the PE protective shell can ensure the survival of the optical fiber during the liner concrete pouring process. In addition, the inner and outer layout of the optical cable is shown in Fig. 16.

#### Layout of vibrational chord strain gauge

**Fig. 17:**
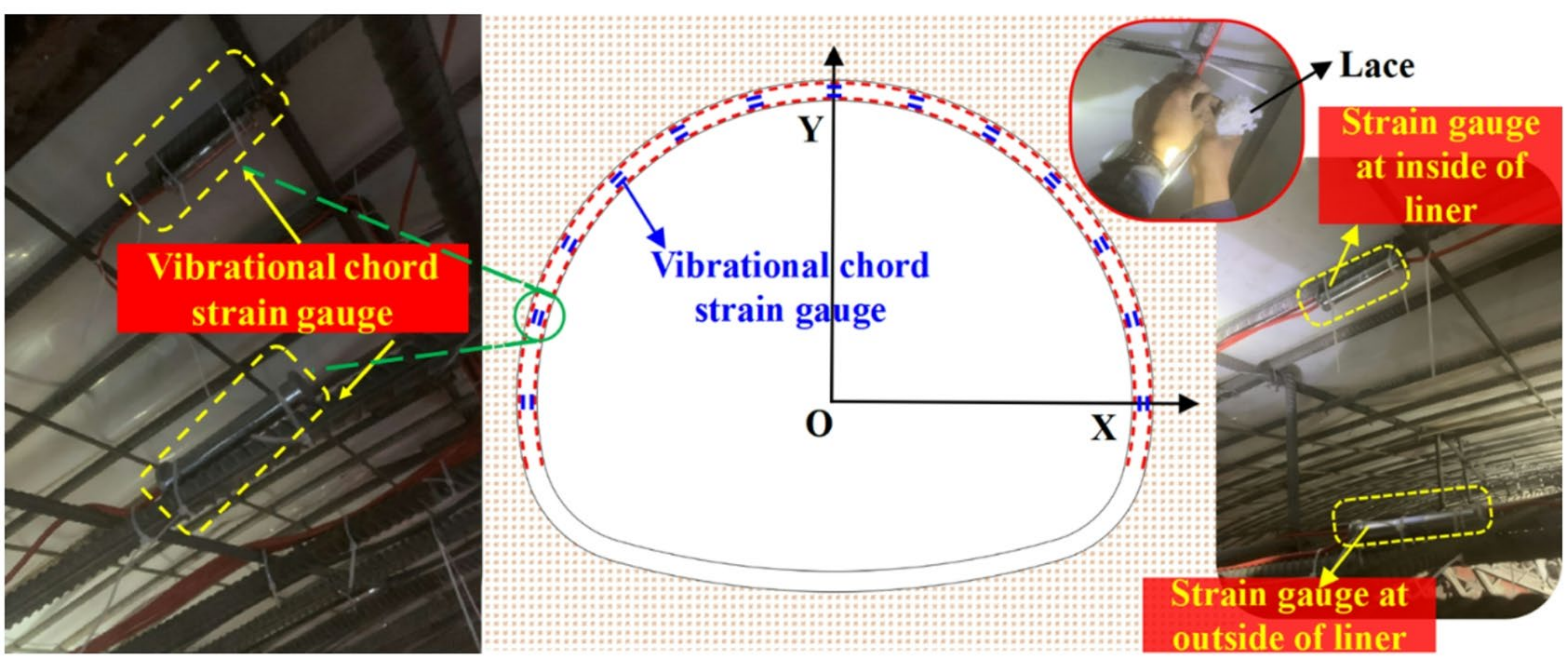
Layout of vibrational chord strain gauge.

To verify the accuracy of the Φ-OTDR transformation formula, vibrational chord strain gauges are installed at 13 positions on the tunnel section according to Fig. 17. Vibrational chord strain gauges are fixed to the main reinforcement with laces after the welding of the secondary liner steel frame is completed. Notably, vibrational chord strain gauges are arranged inside and outside the liner at the same position, which is the same as the distributed optical cable.

### Verification of Φ-OTDR strain monitoring results

**Fig. 18:**
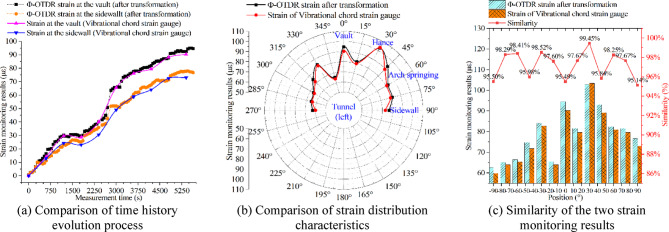
Comparison of monitoring results of Φ-OTDR strain and vibrational chord strain on the outside of liner.

The Φ-OTDR strain is collected once in 1 min through the DSS equipment after the installation of the monitoring instrument is completed. Vibrational chord strain gauges use strain acquisition device to collect strain data every 10 min. A total of 90 min of strain data are collected. The results of the Φ-OTDR strain after the transformation are compared with the vibrational chord strain results as shown in Fig. 18. The comparison of the time history evolution process shows that the evolution trend of the two measured results is similar. The strain results show that the liner tends to be stable after 90 min. From the liner strain distribution results of 90 min, the two measured results are consistent. The similarity analysis of the strain monitoring results shows that the two monitoring results are in good agreement within the 5% error range. In summary, the transformation method of Φ-OTDR strain and tunnel liner strain in this study is feasible, which provides a theoretical basis for Φ-OTDR optical fiber monitoring in tunnels.

## Application of Φ-OTDR optical fiber in tunnel safety monitoring

Although the monitoring on tunnel liner can be realized based on the monitoring principle of Φ-OTDR optical fiber, it is also necessary to serve the safety early warning in tunnel monitoring to ensure the safety of workers. Therefore, this study establishes a safety warning platform for Φ-OTDR optical fiber in tunnel monitoring.

### Safety warning indexes in Φ-OTDR optical fiber monitoring

The current distributed optical fiber strain monitoring is more reliable according to the existing research and the principle of Φ-OTDR optical fiber monitoring. In addition, the safety of tunnel construction mainly includes the safety of initial support and secondary liner. Therefore, the three early warning indexes of initial support stress, secondary liner stress and structural safety factor are proposed to judge the tunnel safety based on the Φ-OTDR optical fiber strain monitoring.

#### Calculus of initial support stress

The distributed optical fiber is arranged on the initial support surface. The arrangement along the longitudinal direction of the tunnel is the same as that of the secondary liner (see Fig. 15). The initial support strain (${\varepsilon _{ini}}$) after the transformation of the Φ-OTDR monitoring results is multiplied by the elastic modulus (${E_{ini}}$) of the initial support concrete to obtain the corresponding initial support stress (${\sigma _{ini}}$), see Eq. (10).

10 $${\sigma _{ini}}={\varepsilon _{ini}} \times {E_{ini}}$$

#### Calculus of secondary liner stress

The structural strain measured on both sides of the secondary liner can be calculated according to Eq. (11) to obtain the stress on both sides of the secondary liner.

11 $$\left\{ \begin{gathered} {\sigma _{in}}={\varepsilon _{in}} \times {E_{liner}} \hfill \\ {\sigma _{out}}={\varepsilon _{out}} \times {E_{liner}} \hfill \\ \end{gathered} \right.$$

where ${\varepsilon _{in}}$ is the inner strain of the secondary liner. ${\varepsilon _{out}}$ is the outer strain of the secondary liner. ${E_{liner}}$ is elastic modulus of secondary liner. ${\sigma _{in}}$ is the inner stress of the secondary liner. ${\sigma _{out}}$ is the outside stress of secondary liner.

#### Calculus of liner structural safety factor

The axial force and bending moment of the secondary liner in the unit length range can be obtained according to the Eqs. (12) and (13).

12 $${N_{liner}}=\frac{{\left( {{\sigma _{in}}+{\sigma _{out}}} \right)}}{2} \times b \times h$$

13 $${M_{liner}}=\frac{{\left( {{\sigma _{in}} - {\sigma _{out}}} \right) \times {h^2} \times b}}{{12}}$$

where ${N_{liner}}$ is the axial force of the liner. ${M_{liner}}$ is the bending moment of the liner. *b* is the unit length, take as 1 m. *h* is the liner thickness.

The safety factor *K* of the liner structure is calculated by Eq. (14) according to the specification^35^.

14 $$\left\{ \begin{gathered} KN_{{liner}} \le 1.75R_{t} bh/\left( {\frac{{6e_{0} }}{h} - 1} \right) \hfill \\ KM_{{liner}} \le R_{w} bx^{'} (h_{0} - \frac{{x^{'} }}{2}) + R_{g} A_{g}^{'} \left( {h_{0} - a^{'} } \right) \hfill \\ \end{gathered} \right.$$

where ${R_t}$ is the ultimate tensile strength of liner. ${R_w}$ is the ultimate compressive strength of the liner. ${R_g}$ is the strength of steel bar. $A_{g}^{\prime }$ is the cross-sectional area of the tensile steel bar. ${h_0}$ is the effective height of the section. ${e_0}$ is the section eccentricity. $x^{\prime}$ is the height of concrete compression zone. $a^{\prime}$ is the distance from the center of gravity of the compressed steel bar to the nearest edge of the section.

### Φ-OTDR optical fiber automatic monitoring and early warning platform

#### Safety threshold of monitoring indexes

The safety threshold of concrete compressive and tensile strength is shown in Table 2 according to the specification^34^.

**Table 2:** Safety threshold of compressive and tensile strength of concrete (unit: MPa).

Strength	Concrete strength grade							
	C15	C20	C25	C30	C35	C40	C45	C50
*f*_*c*_	7.2	9.6	11.9	14.3	16.7	19.1	21.1	23.1
*f*_*t*_	0.91	1.10	1.27	1.43	1.57	1.71	1.80	1.89

The safety threshold of liner structural safety factor is shown in Table 3 according to the specification^35^.

**Table 3:** Safety threshold of liner structural safety factor.

Load combination		Main load	Main load + additional load
Damage reason	The steel bar reaches the calculated strength or the concrete reaches the compressive or shear ultimate strength.	2.0	1.7
	Concrete reaches the ultimate tensile strength.	2.4	2.0

#### Automatic monitoring and early warning platform

**Fig. 19:**
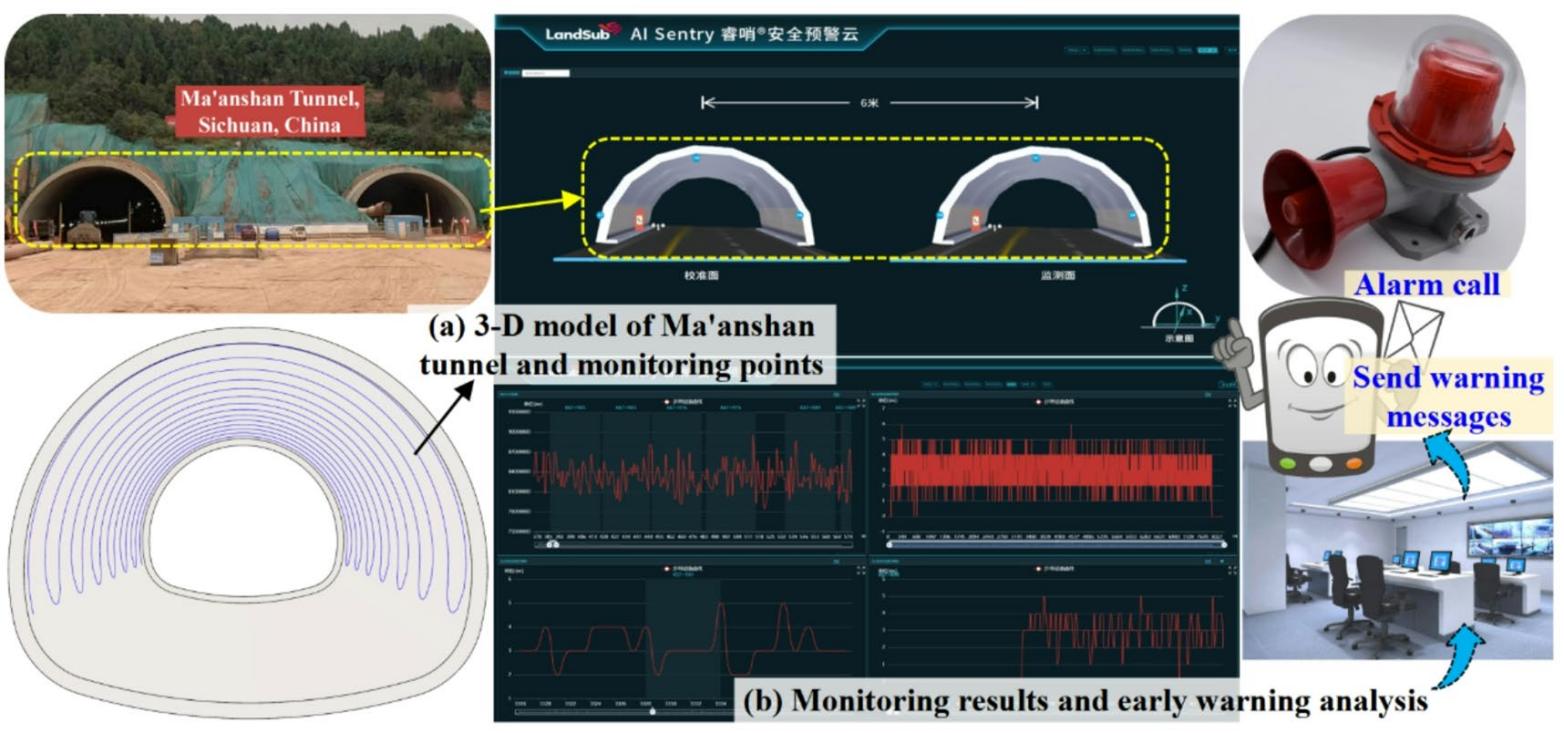
Automatic monitoring and early warning platform.

An automatic monitoring and early warning platform for Ma’anshan tunnel is established on the basis of the above research. Firstly, a 3-D model of Ma’anshan tunnel and monitoring points is established based on BIM technology. Secondly, the strain results measured by Φ-OTDR fiber are transmitted to the platform in time through wireless transmission technology to realize automatic monitoring. Thirdly, the transformation formula, structural stress calculus and liner safety factor calculus are implanted into the platform by program coding. Finally, the monitoring index safety threshold library is imported into the platform. The platform sends warning messages to the workers’ mobile phone in time and triggers the siren of the monitoring room when the monitoring results exceed the safety threshold, thus intelligently ensuring the tunnel construction safety. The automatic monitoring and early warning platform developed by Chengdu Land-Sub Technology Co., Ltd. is shown in Fig. 19.

### Analysis of Φ-OTDR optical fiber automatic monitoring results

**Fig. 20:**
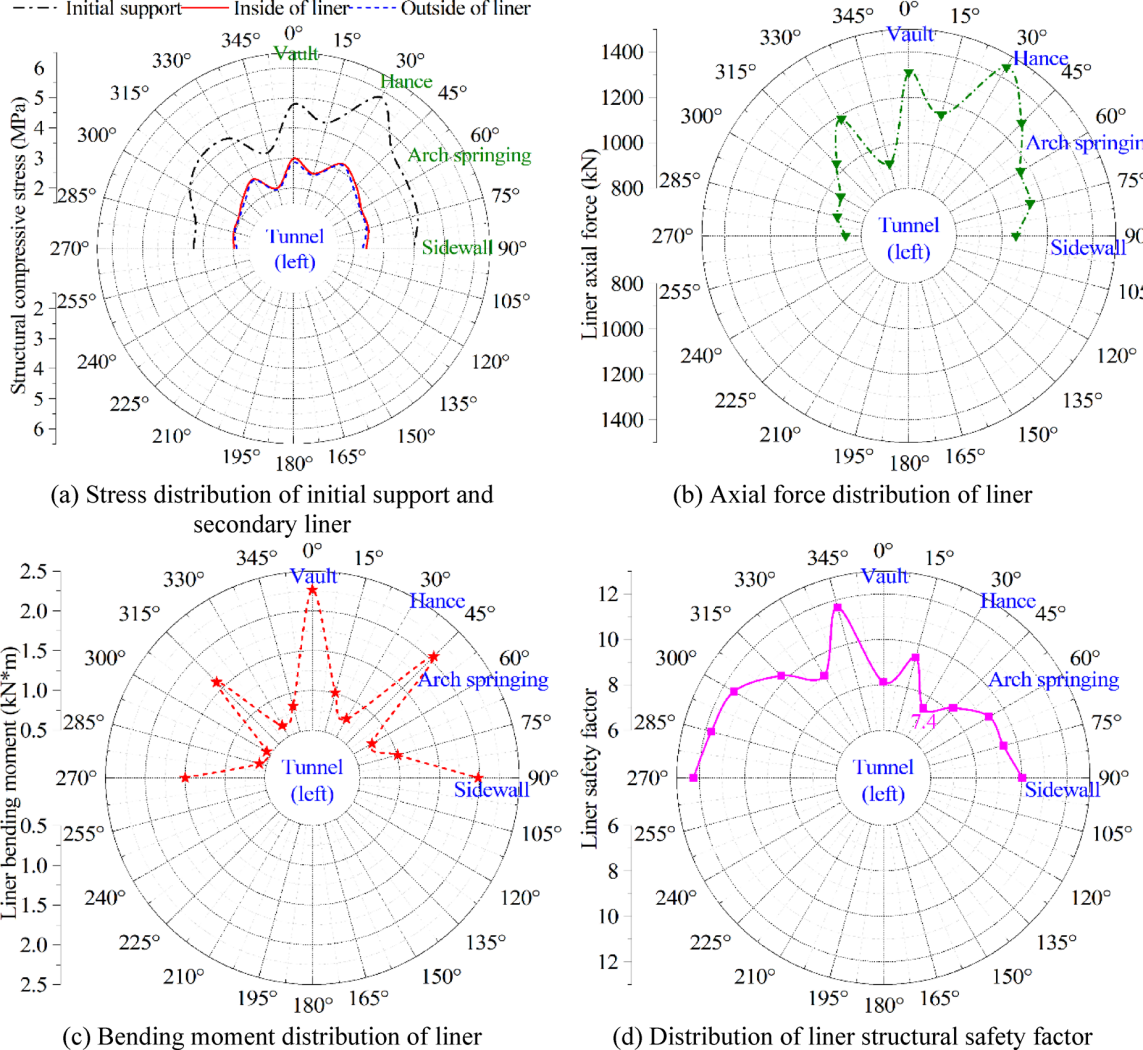
Φ-OTDR optical fiber automatic monitoring results.

After two months of automatic monitoring by Φ-OTDR optical fiber in Ma’anshan tunnel, the strain results of the initial support and the secondary liner are extracted. Through the intelligent analysis of the monitoring platform, the results of each early warning index of the initial support and the secondary liner are shown in Fig. 20. The results indicate that tunnel construction exerts a more pronounced effect on the stress of the initial support than on that of the secondary liner. This is expected, as the initial support directly bears the stress redistribution caused by excavation and temporary construction loads, while the secondary liner primarily serves as a long-term load-bearing and stability-enhancing structure. Despite these construction-related fluctuations, the maximum stress in the initial support remains below the allowable limit of 11.9 MPa, demonstrating that the support system is operating within a safe stress range. The stress, axial force and bending moment of the tunnel (left) liner near the right tunnel are relatively large, as this region is subjected to the combined effects of vertical overburden pressure and horizontal surrounding rock pressure. The structural safety factor distribution (Fig. 20d) reflects the interaction of these internal forces. The minimum safety factor is observed at the tunnel right hance, with a value of 7.4. Although this location represents the most unfavorable condition within the monitored section, the safety factor remains well above the design requirement of 2.4, indicating a considerable safety margin. At other locations, higher safety factors are obtained, further confirming that the overall load-bearing performance of the tunnel is stable and well within the design capacity.

## Conclusion

This research developed a DSS monitoring system based on Φ-OTDR sensing technology. To apply the system to tunnel safety monitoring, the transformation method of Φ-OTDR optical fiber strain and tunnel liner strain is proposed, and a tunnel automatic monitoring and early warning platform is established. The main conclusions are as follows:

1. A distributed optical fiber stress and strain monitoring system (DSS) is developed based on the Φ-OTDR strain sensing mechanism. The system is equipped with GYTS distributed armored optical cable. The loose sleeve in the cable has good toughness and high strength, making the GYTS optical cable survival rate of 92%.
2. The transformation method between Φ-OTDR strain and liner strain is proposed, and the dynamic and static tests of straight concrete beams are used to calibrate the transformation relationship between Φ-OTDR strain and structural strain. The test results show that the strain increases linearly with the increase of test load and time. Further, the development trend of Φ-OTDR strain accumulation over time under the influence of the external environment is $\varepsilon _{{fiber}}^{t}=(0.023 \pm 0.001) \cdot t+(0.1425 \pm 1.1795)$ through the static test results. Combined with the dynamic test results, the transformation relationship between Φ-OTDR strain and structural strain is ${\varepsilon _{struc}}=1.263 \cdot {\varepsilon _{fiber}} - 0.029 \cdot t+0.708$.
3. The optical fiber is arranged in U shape and fixed to the main reinforcement with laces in the tunnel safety monitoring. Notably, the tunnel field test shows that the coincidence between the converted Φ-OTDR strain results and the vibrational chord strain results reaches 95%, which verifies the accuracy of the Φ-OTDR strain and liner strain transformation method proposed in this study and the feasibility of Φ-OTDR fiber in tunnel structural strain monitoring.
4. A safety warning platform for tunnel monitoring is established based on Φ-OTDR optical fiber technology. The platform uses the three early warning indexes of initial support stress, secondary liner stress and structural safety factor are proposed to judge the tunnel safety. Notably, the optical fiber on the liner is arranged on both sides. Moreover, the measured Φ-OTDR strain is presented as stress, axial force, bending moment and safety factor by implanting the calculation program of the early warning index. The platform sends warning messages to the workers’ mobile phone in time and triggers the siren of the monitoring room when the monitoring results exceed the safety threshold, thus intelligently ensuring the tunnel construction safety.

## Data Availability

The datasets used and/or analysed during the current study are available from the corresponding author on reasonable request.
